# Mechanistic Approach of Nano Carriers for Targeted in Cancer Chemotherapy: A Newer Strategy for Novel Drug Delivery System

**DOI:** 10.3390/polym14122321

**Published:** 2022-06-08

**Authors:** Niladri Shekhar Dey

**Affiliations:** Jiaganj Institute of Pharmacy, Jiaganj, Murshidabad 742123, India; niladrisekhar111@gmail.com

**Keywords:** nanolipid vesicles, specific antibody, cellular apoptosis, breast cancer chemotherapy

## Abstract

The application of nanomedicine represents an innovative approach for the treatment in the modern field of cancer chemotherapy. In the present research work, tamoxifen citrate loaded nanolipid vesicles were prepared conjugated with phosphoethanolamine as the linker molecule, and the specific antibody was tagged with the linker molecule on the bilayer surface of the vesicles. The main objective of this study is to determine the efficacy and biological behavior of antibody conjugated nanoliposome in breast cancer cell lines. Percentage of drug loading and loading efficiency was done and their results were compared to theoretical drug loading. The average diameter of those vesicles was within the 100 nm range, which is revealed in FESEM and TEM images and their lamellarity was observed in cryo-TEM images. The hydrodynamic diameter was done by particle size analysis and the surface charge was determined by the zeta potential parameter. Predominant cellular uptake was observed for antibody conjugated nanolipid vesicles in MCF-7 and MDA-MB-453 human breast cancer cell lines. A cellular apoptosis assay was conducted by flow cytometer (FACS). All experimental data would be more beneficial for the treatment of breast cancer chemotherapy. Further studies are warranted to investigate the efficacy and safety of antibody conjugated nanolipid vesicles in vivo for breast cancer animal model.

## 1. Introduction

In the modern field of oncology, chemotherapy has been reported to have potential benefits to arrest cancerous cell proliferation, neovascularisation, metastasis in tumor cells as well as the capability to control the growth rate of malignant cells other than any harmful effect of normal healthy cells [1]. Lipid bilayer vesicles are spherical in shape and are constructed with phospholipids and cholesterol, with an aqueous internal compartment. The design and fabrication of such a type of drug delivery showed an important role in their low toxicity, flexibility, biocompatibility and biodegradability in the pharmaceutical field [2]. Different anticancer drug-loaded nanocarriers have been applied in cancer chemotherapy for passive as well as active targeting purposes. Compared to other nanoformulations, lipid vesicles in nanosize dimensions showed potential advantages due to their pharmacokinetic and pharmacodynamic activities. Further, this type of novel drug delivery system reported great advantages to improve solubility properties in case of very poor soluble or sparingly soluble drugs [3]. Bilayer lipid vesicles in a nanoscale range, which are also known as nanoliposomes, are becoming more popular for their quick action and safety as well as their controlled release properties, and also have a better biocompatibility index for newer strategies in the field of oncology [4]. Polymeric nanovesicles are quite similar to liposomes which are formed by amphiphilic polymers in aqueous solutions and can be entrapped or loaded with anticancer drug molecules via electrostatic or hydrogen bonding interactions. Polymeric nanolipid vesicles can be applied for active targeting by tagging with specific ligands, such as antibodies, folic acid and peptide molecules to control the abnormal growth of malignant cells [5]. Targeted drug delivery technology promotes entrapment efficiency as well as improves long circulation in different organs of the human body [6]. The application of specific ligand molecules, such as antibodies, by tagging with bilayer vesicles of nanocarriers can more specifically recognize malignant cells [7]. The traditional application of nanoformulations loaded with anticancer drug(s) can affect malignant cells through their “enhanced permeability and retention effect” (EPR) by the passive targeting. The active targeting by conjugation with antibody or aptamar to the surface of nanosized carriers improves the therapeutic efficacy of chemotherapies through the selective targeting of tumor tissues [8].

The loading of an active pharmaceutical ingredient (API) in a liposomal bilayer surface or within the core of lipid vesicles nowadays is the main objective for targeted drug delivery. For avoiding the risk factors of drug delivery and improving drug release patterns as well as pharmacokinetic parameters, novel drug delivery was established by using nanoliposomes. Several assays were applied to demonstrate the quality and safety of bioequivalence tests for liposomal formulations [9].

In previous work, we have designed tamoxifen citrate (TC) loaded nanoliposomes conjugated with phosphoethanolamine (PE) by a thin-film hydration method and their characterization such as, surface morphology, in vitro drug release and in vivo pharmacokinetics, cell viability, results have been studied. [10].

The citrate salt of the antineoplastic drug tamoxifen which is a nonsteroidal selective estrogen receptor modulator (SERM). Tamoxifen competitively inhibits the binding of estradiol to estrogen receptors, thereby preventing the receptor from binding to the estrogen-response element on DNA. The result is a reduction in DNA synthesis and cellular response to estrogen. In addition, tamoxifen up-regulates the production of transforming growth factor B (TGFb), a factor that inhibits tumor cell growth, and down-regulates insulin-like growth factor 1 (IGF-1), a factor that stimulates breast cancer cell growth [11,12].

In the present work, tamoxifen citrate loaded nanolipid vesicles conjugated with phosphoethanolamine (linker molecule) were developed, and a specific antibody was tagged with linker molecule on the bilayer surface of nanolipid vesicles. The biological behavior of those formulations was evaluated in MCF-7 as well as the MDA-MB-453 human breast cancer cell lines. The lamellarity of the vesicular structure was studied by cryo-TEM images. Cellular apoptosis of modified lipid vesicles was conducted by FACS analysis.

## 2. Materials and Methods

### 2.1. Materials

Tamoxifen citrate was obtained from the Arex Pharmaceutical laboratory, Mumbai, India, as a gift sample, and MCF-7 and MDA-MB-453 (human breast cancer cell line) were obtained from the Chittaranjan National Cancer Research Centre Hospital (Kolkata, India) and Indian Institute of Chemical Biology (Kolkata, India), respectively.

Soya-L-α-lecithin, (2-hydroxypropyl)-β-cyclodextrin, dialysis bag (membrane-60) and fluorescein isothiocyanate (Isomer I) were procured from HiMedia Laboratories Pvt. Ltd. (Mumbai, India). Other chemicals, such as cholesterol (Merck, Mumbai, India), 1,2 distearoylsn-glycero-3-phosphoethanolamine (Sigma-Aldrich, Bangalore, India), butylated hydroxy toluene (Qualigens Fine Chemicals, Division of Glaxo India Limited, Mumbai, India), chloroform (Merck, Mumbai, India), Dulbecco’s Modified Eagle’s Medium and 3-[4,5-dimethylthiazol 2-yl]-2,5-diphenyltetrazolium bromide (Sigma-Aldrich, Bangalore, India), FITC anti-human CD 340 (erbB2/HER-2) antibody and Annexin V-FITC staining kit were purchased from BioLegend, Inc., San Diego, CA, USA. All the other chemicals used were of analytical grade.

### 2.2. Method

#### 2.2.1. Procedure of Tamoxifen Citrate Loaded Nanolipid Vesicles and Tamoxifen Citrate Loaded Nanolipid Vesicles Tagging with Antibody

Nanolipid vesicles were prepared by the lipid layer hydration technique [13,14]. The required quantity of soya lecithin, cholesterol and beta hydroxy toluene (BHT) (1% *w*/*v*) and the anticancer drug, tamoxifen citrate, were taken in a round bottom flask and dissolved in an organic solvent, such as chloroform, with continuous stirring. The flask was attached with a rotary vacuum evaporator (Rotavap, model PBU-6, Superfit Continental Pvt. Ltd., Mumbai, India) fitted with an A3S aspirator (Eyela, Rikakikaic, Ltd., Taguig City, Philippines). The round bottom flask was rotated at 125 rpm at 40 °C in a water bath until the complete evaporation of chloroform. On the next day, the flask was fitted with a rotary vacuum evaporator in a water bath rehydrated with phosphate buffer saline (pH 7.4) at 60 °C until the lipid film was dispersed in the aqueous phase. The round bottom flask containing the dispersion was kept in a bath-type sonicator (Trans-o-sonic, Mumbai, India) at a frequency of about 30 ± 3 KHz at room temperature (25 °C) for 1 h. The flask was kept at 25 °C for about 1 h, after sonication, for vesicle formation and then the flask was kept overnight at 4 °C. The next day, multilamellar lipid vesicles were separated from the liposomal suspension by centrifugation (3K30 Sigma Lab Centrifuge, Merrington Hall Farm, Shrewsbury, UK) at 5000 rpm at 4 °C for 10 min. Then, the supernatant of the vesicular suspensions was re-centrifuged at 15,000 rpm at 4 °C for 1 h to obtain nanosized lipid vesicles [15]. The nanoformulation was separated and collected in a Petri dish and the formulation was kept in a laboratory freeze dryer (Instrumentation India Ltd., Kolkata, India) for lyophilization. For the preparation of phosphoethanolamine conjugated nanolipid vesicles, the required amount of drug (tamoxifen citrate), cholesterol, soya lecithin, BHT and phosphoethanolamine (PE) were dissolved in chloroform with vigorous shaking. The remaining procedures were similar to what was described earlier. The hydroxyl group (-OH group) of phosphoethanolamine (PE) forms conjugation with the phosphate group of soyalecthin (phospholipid) upon incubation at room temperature [14,16].

#### 2.2.2. Preparation of Antibody Conjugated Liposome

The antibodies were conjugated to the phosphoethanolamine (PE) group present on the phospholipid bilayer surface by creating a thiol-reactive phospholipid derivative. To 1 mL of nanolipid vesicular (TNL-PE) suspension suspended in PBS, pH 7.2, 25 mL of 20 mM *N*-hydroxylsuccinimydyl 3-(2-pyridyldithio) propionate (SPDP) was added, and the reaction was carried out for 30 min [16,17]. One hundred microliters of FITC anti-human CD340 (erbB2/HER-2) antibody were incubated for reaction with 25 mL of SPDP for 30 min [18]. Twelve milligrams of dithiothreitol (DTT) were added and kept for 30 min at room temperature. The antibody-containing fraction was subjected to gel filtration (with phosphate-buffered saline, pH 7.2), and the reaction was carried out with the vesicular suspension overnight. Conjugation was initiated by mixing lipid vesicles with thiolated antibody [19]. After overnight conjugation, the nanolipid vesicles were separated from unbound protein by centrifugation [18].

#### 2.2.3. Determination of Monoclonal Antibodies on the Surface of Nanoliosomes

A secondary antibody, FITC-labeled sheep anti-human immunoglobulin, was used to identify the presence of monoclonal antibody, such as anti-human CD340 (erbB2/HER-2) antibody on the surface of the nanoliposomes. Monoclonal antibody-modified nanoliposome or nanolipid vesicles were treated with 0.5 μL of labeled sheep anti-human immunoglobulin for 2 h at room temperature. The volume ratio between the solution of secondary monoclonal antibodies and dispersion of immuno-nanocarriers was 1:1000. Samples were centrifuged at 10,000 rpm for 15 min and washed twice with 1 mL of phosphate-buffered saline (PBS, pH 7.4) to eliminate the excess antibody. After a final washing, the sediment of the sample solution was redispersed in PBS (pH 7.4), and the fluorescence intensity of fluorescent dye (fluorescein, λ_max_ 494 nm and λ_max_ 525 nm) was measured using a microplate reader (Safire2™, Tecan, Switzerland). The fluorescence intensity was compared with that of the noncoated nanolipid vesicles, FITC-labeled sheep anti-human immunoglobulin, and phosphate-buffered saline at pH 7.4 as the medium and the wavelength was revealed by flow cytometer [20].

#### 2.2.4. Field Emission Scanning Electron Microscopy (FESEM)

Field emission scanning electron microscopy (FESEM, JEOL JSM 6700 F Tokyo, Japan) was applied for the evaluation of the surface morphology of nanosized lipid vesicles. The double-sided adhesive tapes stubs were mounted for lyophilized lipid vesicular formulation, then vacuum coated with platinum or gold using the JEOL JFC 1600 autofine coater (JEOL, Tokyo, Japan). The formulations were then kept for evaluation under the field emission scanning electron microscope [21].

#### 2.2.5. Transmission Electron Microscopy (TEM)

The morphology of nanolipid vesicles was detected using a transmission electron microscope [FEI (Type FP 5018/40) Tecnai G2 Spirit Bio TWIN]. The formulations were dispersed in de-ionized water at a concentration of 500 μg/mL. A drop of the sample was placed onto a 300-mesh copper grid coated with carbon to measure the morphology and size distribution of nanolipid vesicles. The grid was air-dried overnight to remove surface water at room temperature. The TEM image was obtained on the transmission electron microscope operating at an accelerating voltage of 100 kV [22].

#### 2.2.6. Cryo-Transmission Electron Microscopy (Cryo-TEM)

The sample of nanovesicular formulation was prepared prior to vitrification for cryo-electron microscopic analysis at room temperature. The lyophilized formulation of drug-loaded vesicles (1 mg) was suspended in 2 mL of double-distilled water. Then, the aqueous suspension was vortexed in a cyclomixer for 15 min, and to prevent agglomeration, we have sonicated the sample for 5–7 min at room temperature in a water bath. Samples were preserved by vitrification and supported by quantifoil holey carbon films on copper grids. Vitrified samples were prepared by applying 4 μL drops of sample suspension to a clean grid, blotting away with filter paper, and immediately plunging into liquid ethane. Grids were kept in liquid nitrogen until they were transferred to the electron microscope for imaging. Cryo-TEM images were taken using electron microscopy (Tecnai Polara G2, FEI Company, Eindhoven, Netherlands), operating at 300 kV equipped with an FEI Eagle 4 K × 4 K charge-coupled device (CCD) camera. Vitreous ice grids were transferred under the electron microscope using a cryo stage, which maintains the grids at a temperature below −170 °C. Images of each grid were taken to assess the overall distribution of the specimen [23,24].

#### 2.2.7. Energy Dispersive X-ray Analysis (EDX Analysis)

The EDX technique was applied to recognize the elemental composition of the sample or an area of the specimen. The EDX analysis system works as an integrated feature of a scanning electron microscope (SEM) (JSM-6360, JEOL, Tokyo, Japan) [25].

#### 2.2.8. Evaluation of Drug Loading

The required amount of nanolipid vesicles (2 mg) was properly dissolved in ethanol with vigorous shaking. The sample was centrifuged at 10,000 rpm for 2 min and the absorbance of the supernatant sample was measured at 238 nm using a UV/VIS spectrophotometer (Model Intech-295, Gentaur GmbH, Aachen, Germany). The same procedure has been conducted for nanolipid vesicles without the drug. All the experimental results for drug loading were carried out in a triplicate manner. The absorbance of the drug was measured as the difference between the absorbance readings obtained from the vesicle preparation with the drug and without the drug to avoid any minor error due to the excipients. The % loading and the % loading efficiency were calculated using the following formula [26,27].

% Loading = (Amount of tamoxifen citrate in nanoliposomes/Amount of nanoliposome obtained) × 100%

% Loading efficiency = (Actual amount of drug loaded in nanoliposomes/Theoretical amount of drug loaded in nanoliposomes) × 100

#### 2.2.9. Particle Size and Zeta Potential Measurement

Particle sizes and surface charges were determined by dynamic light scattering and laser doppler, respectively, using a Zetasizer Nano and analyzed using Data Transfer Assistance (DTS) software (Malvern Instrument, Malvern, UK) [28].

#### 2.2.10. In Vitro Cellular Uptake of Nanolipid Vesicles

The human breast cancer cell lines were applied for the visualization of cellular uptake of nanolipid vesicles by confocal laser scanning microscopy. The human breast cancer cell lines MCF-7 cells (estrogen receptor-positive) and MDA-MB-453 cells (estrogen receptor-negative) were cultivated for 24 h on the top of coverslips in six-well culture plates (3 mL/well at a density of 10^4^ cells/mL). The cell lines MCF-7 and MDA-MB-453 were obtained from the Chittaranjan Cancer Research Institute, Kolkata and the Indian Institute of Chemical Biology, Kolkata, respectively. Antibody conjugated nanolipid vesicles were then applied at a concentration range of 50 μg/mL and 100 µg/mL in the MCF-7 and MDA-MB-453 cell culture medium. Cells were co-incubated with FITC loaded nanolipid vesicles without the drug as an additional control. Both the cancer cell lines were washed thoroughly for three hours of incubation and then fixed with a paraformaldehyde aqueous solution of 4% (*v*/*v*). After fixing both cell lines for 15 min, they were rinsed with phosphate buffer saline (pH 7.4). For the next step, the coverslips were placed carefully so that both the cell lines adhered to the coverslips upon the slide. The slides were dried and kept under the confocal laser scanning microscopy (Andor Spinning Disc Confocal Microscope, Andor Technology, Ireland, UK) [29].

#### 2.2.11. Apoptosis Assay by FACS Analysis

MCF-7 cells were cultured and divided into control and test groups. Normal cells were treated with a serum-free medium for 24 h. Double-staining provided the percentage of live, apoptotic, and necrotic cells. Apoptotic cells were recognized by binding with fluorescein-labeled annexin V to exposed phosphatidylserine on the cell surface and necrosis by staining with propidium iodide (Texas Red). Cell clusters were identified as described in the annexin V-FITC staining kit (BioLegend, Inc., San Diego, CA, USA) according to the manufacturer’s recommendations with flow cytometric analysis. The cells were incubated with various concentrations (50 and 100 μM) of the nanoliposomal formulation (TNL-PE) for 5 h [28]. After trypsinization, cells were harvested by centrifugation at 3000 rpm for 5 min. They were then resuspended at 10^6^ cells/100 μL in a binding buffer, stained with annexin V incubation reagent (1 μL annexin V-FITC (25 μg/mL), 10 μL binding buffer, 10 μL propidium iodide (PI) (50 μg/mL), and 79 μL H_2_O) and incubated in the dark for 15 min at room temperature [27]. The cells (10,000 cells per sample) were immediately analyzed with the addition of 500 μL of the binding buffer by using a FACScan flow cytometer (SFAC Calibur, BD Biosciences, San Jose, CA, USA). Quadrant markers were applied to identify the four cell populations (annexin V-/propidium iodide+, annexin V+/propidium iodide+, annexinV-/propidium iodide-, annexin V+/propidium iodide-). The results were expressed as a percentage of the gated events [29].

#### 2.2.12. Statistical Analysis

All experimental data were conducted in a triplicate manner for checking the reproducibility. For three experimental results, each value was reported as mean ± standard deviation (SD). Statistical calculations for all data were performed using the GraphPad Prism^TM^ (version 5.0) software (San Diego, CA, USA). In each phase, the probability value (*p*) < 0.05 was accepted as statistically significant.

## 3. Results

### 3.1. Surface Morphology

**Figure 1:**
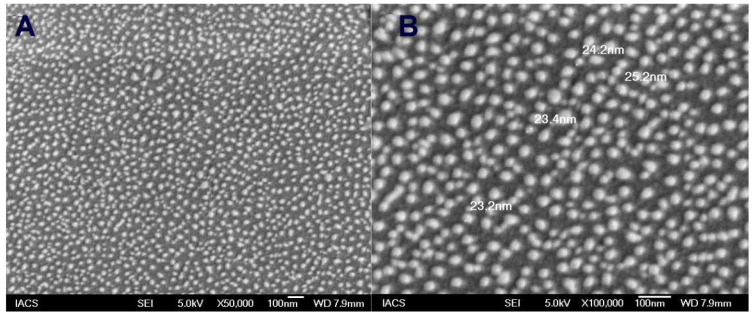
FESEM images of (**A**) tamoxifen citrate loaded nanolipid vesicles; (**B**) antibody conjugated tamoxifen citrate-phosphoethanolamine nanolipid vesicles.

FESEM photographs of tamoxifen citrate loaded nanolipid vesicles (Figure 1A) and antibody conjugated nanolipid vesicles (Figure 1B) revealed that the vesicular diameter was within 100 nm in their lyophilized state. Both lipid vesicles (Figure 1A,B) had a smooth surface with a spherical shape and they were scattered uniformly in their freeze-dried state. Conjugation of antibody with PE as a linker molecule on modified nanolipid vesicles was found to have little enhancement of the diameter of the lipid vesicles (Figure 1B).

**Figure 2:**
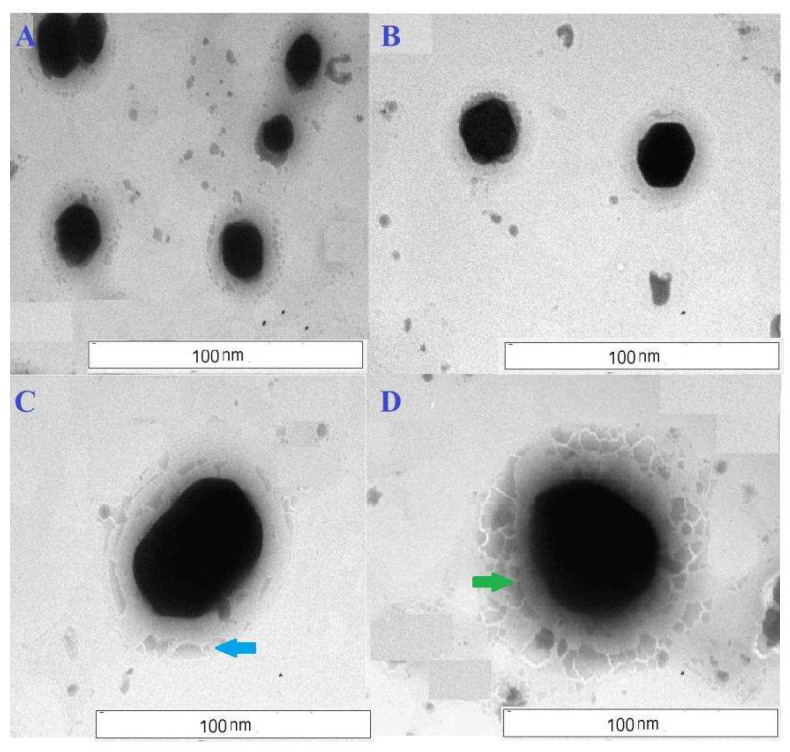
TEM images of (**A**,**C**) tamoxifen citrate loaded nanolipid vesicles; light peripheral demarcation indicates the phospholipid bilayer (shown by sky blue arrow). (**B**,**D**) antibody conjugated tamoxifen citrate-phosphoethanolamine nanolipid vesicles; the presence of antibody with phosphoethanolamine tagging in phospholipid bilayer revealed darker, thicker, polygonal particulate matters (shown by green arrow).

The morphological images of nanoliposomes are shown in the TEM photographs (Figure 2A,B) where the nanoliposomes can easily be identified as discrete vesicles. The black portion indicates the drug that is loaded inside the vesicles (Figure 2A–D) and the light peripheral demarcation indicates the phospholipid bilayer, which contains cholesterol, visible as darker elongated spots (Figure 2C). In the case of antibody conjugation with phosphoethanolamine (PE) in the bilayer surface, the presence of antibody with PE was predominantly visible in numbers of darker, thicker, polygonal particulate matters (Figure 2D).

**Figure 3:**
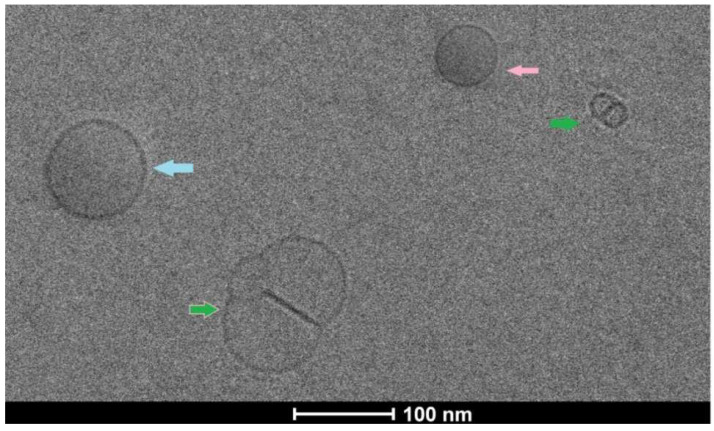
Cryo-TEM images: pink color and sky blue color arrow indicate small unilamellar vesicle (near about 50 nm in diameter) and unilamellar vesicle (near about 100 nm in diameter), respectively. Green color arrow represents two unilamellar vesicles fused with each other.

The cryo-TEM images (Figure 3) revealed the vesicular lamellarity as well as good intactness of antibody conjugated drug-loaded nanocarriers. A few unilamellar spherical vesicles within 100 nm ranges with a smooth surface, and no leakage were found in the cryo-TEM images. Nanolipid vesicles were surrounded by lipid bilayer with anticancer drug loaded within the core, which was indicated by a dark area. However, few vesicles were fused together; represent rigid intactness, and no leakage was observed. The size of the spherical vesicles represents one smaller (50 nm) and near about 100 nm of the vesicles. Here, freeze-dried lipid vesicles were suspended in an aqueous solution for a longer time prior to the cryo-TEM image investigation compared to the particle size analysis. Therefore, there was a predominant uptake of water by the vesicles and simultaneously they were swollen and the hydrodynamic diameter was a little bit enhanced.

**Figure 4:**
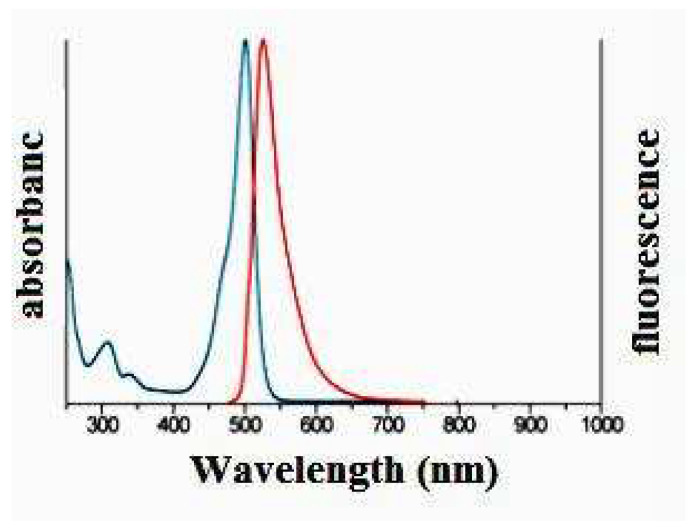
Fluorescence intensity of TNL-PE nanolipid vesicles (blue color wavelength); fluorescence intensity of TNL-PE-Ab nanolipid vesicles (red color wavelength).

The shifting of wavelength for TNL-PE and TNL-PE-Ab showed different fluorescent color intensities (blue to red) in two absorption maxima (λ_max_ 494 nm and λ_max_ 525 nm) by flow cytometer (Figure 4). The results indicated that antibody was labeled in modified nanolipid vesicles (TNL-PE-Ab) by red fluorescence color intensity.

**Figure 5:**
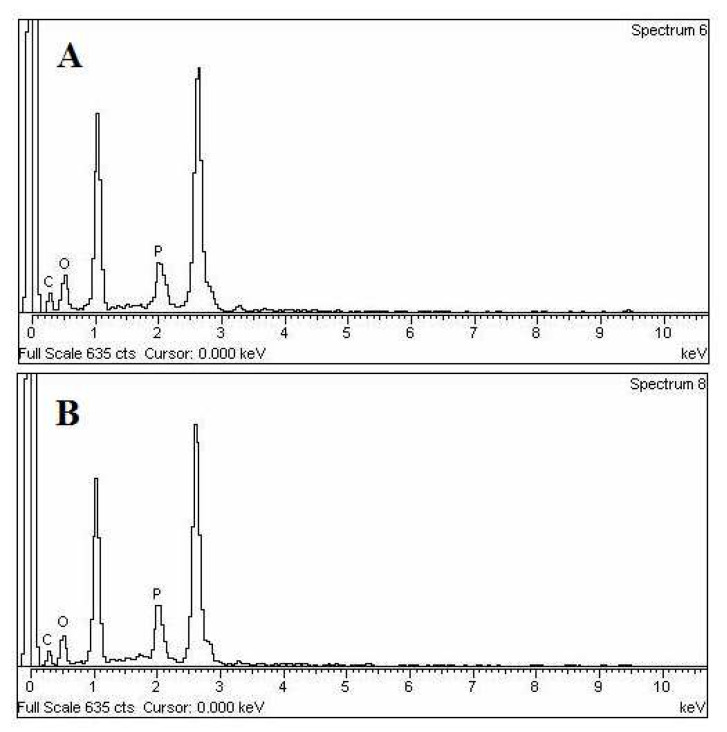
Energy dispersive X-ray (EDX) of (**A**) TNL-PE and (**B**) TNL-PE-Ab.

EDX data (Figure 5 and Table 1) show the weight % and atomic % of different elements (C, O, and P) in the nanolipid vesicles. The weight percentages of C, O, and P in TNL-PE were 37.58, 51.45 and 10.97, respectively, and the values for TNL-PE-Ab was 34.04, 49.03 and 16.92, respectively. The atomic percentages of C, O, and P in TNL-PE were 46.71, 48.00 and 5.29, respectively, and in TNL-PE-Ab, they were 43.97, 47.55 and 8.48, respectively. The proportional differences in weight percentage and atomic percentage values in TNL-PE as compared to TNL-PE-Ab might be due to the incorporation of antibody in tamoxifen citrate loaded nanolipid vesicles. In the figure, the two dominant peaks are sodium and potassium, respectively. The source of sodium and potassium are mainly from the soya lecithin sample, which contained those two element salts as contaminants.

**Figure 6:**
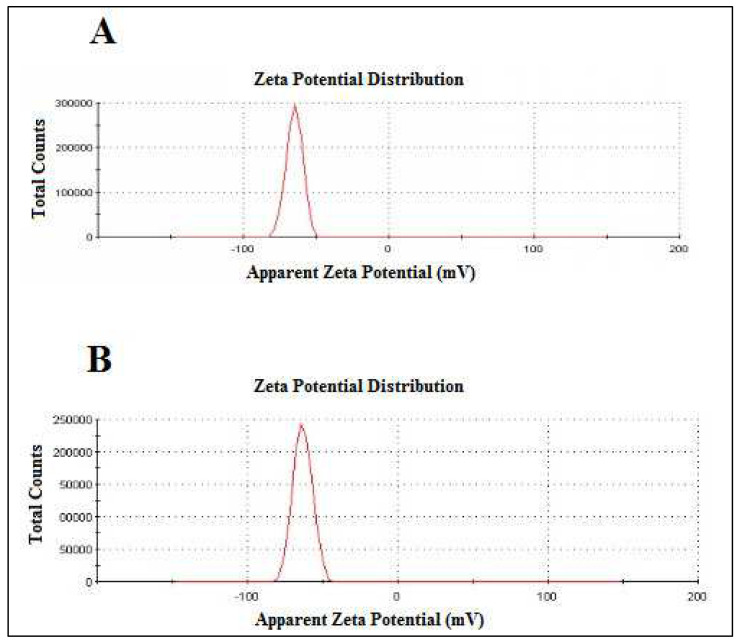
Zeta potential data: (**A**) tamoxifen citrate loaded nanolipid vesicles; (**B**) antibody conjugated tamoxifen citrate loaded nano lipid vesicles.

The surface charge(zeta potential) of TNL-PE and TNL-PE-Ab formulation revealed that due to conjugation of antibody in modified nanolipid vesicles (TNL-PE-Ab) slight positivity compared to nonconjuagated vesicle i.e., TNL-PE and their mobility and conductivity also indicated (Figure 6 and Table 2).

Percentage of drug loading of TNL-PE and TNL-PE-Ab were 3.13 ± 0.08 and 3.46 ± 0.58 respectively, which was compared with entage of perctheoretical loading and their loading efficiency were 90.97 ± 2.34 and 90.13 ± 15.25 respectively (Table 3).

**Figure 7:**
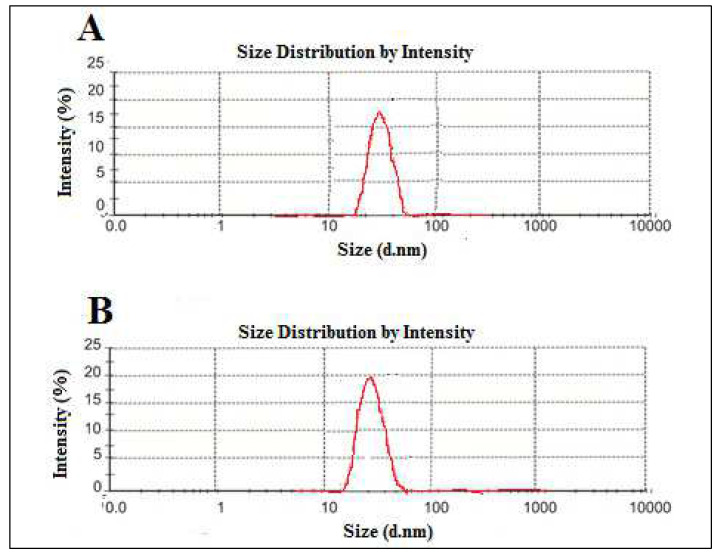
Particle size analysis: (**A**) tamoxifen citrate loaded nanolipid vesicles, (**B**) antibody conjugated tamoxifen citrate loaded nanolipid vesicles.

The z-average values (average diameter) of TNL-PE and TNL-PE-Ab formulation were found to be 51.2 ± 1.50 and 62.3 ± 1.25 d.nm respectively (Figure 7).

### 3.2. Cellular Uptake

**Figure 8:**
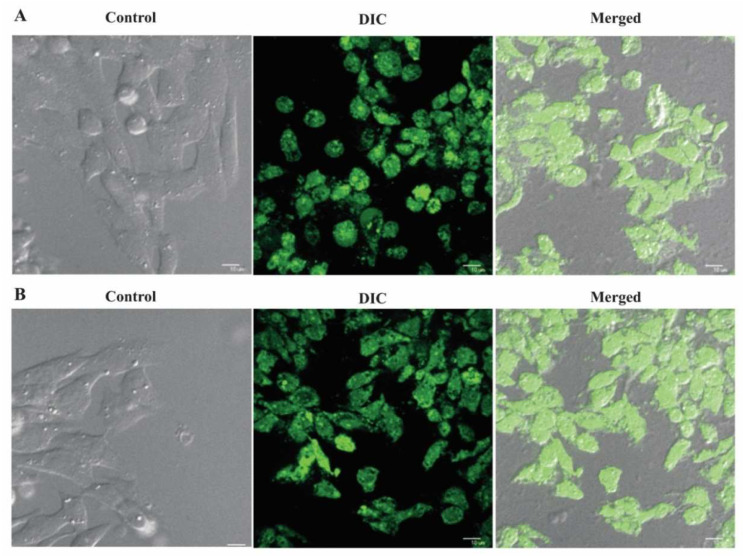
In vitro cellular uptake of antibody conjugated nanolipid vesicles in MCF-7 breast cancer cells. (**A**) Formulation at concentration 50 µg/mL for 1 h; (**B**) formulation at concentration 100 µg/mL for 2 h.

In vitro cellular uptake, a study was also conducted for tamoxifen citrate loaded nanoliposomes conjugated with antibody (FITC anti-human CD 340 (erbB2/HER-2)) in MCF-7 and MDA-MB-453 breast cancer cells at different concentrations, such as 50 and 100 µg/mL for 1 and 2 h, respectively. Figure 8A,B revealed that the vesicles penetrated the cell membrane which was assessed using confocal microscopy images.

Confocal fluorescence microscopy studies were performed to visualize the internalization and subsequent intracellular distribution of antibody (FITC anti-human CD 340 (erbB2/HER-2)) labeled nanolipid vesicles. Cells were incubated with antibody conjugated nanoliposome and remained largely at or near the cell surface accompanied by some cytoplasmic localization, with rapid endocytosis. It has been shown that cellular accumulation of the targeted nanolipid vesicles was faster and had higher accumulation at different time points in different concentrations.

**Figure 9:**
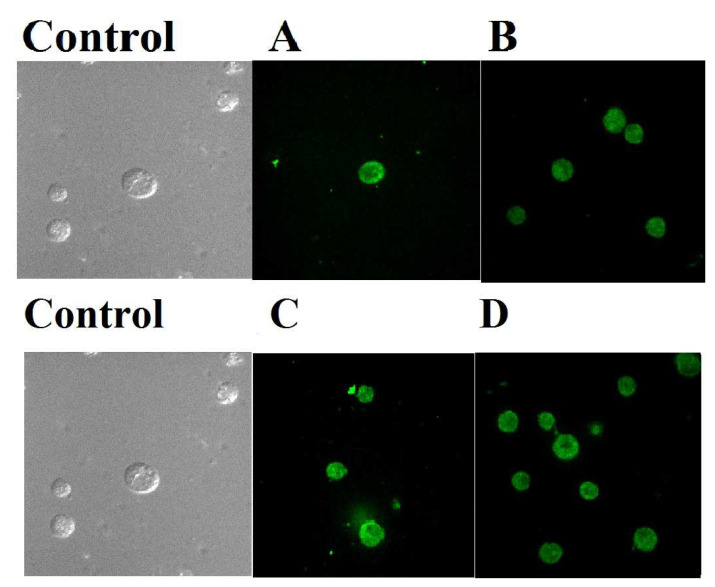
(**A**) Antibody conjugated nanoliposome formulation at concentration 50 µg/mL for 1 h. (**B**) Antibody conjugated nanoliposome formulation at concentration 100 µg/mL for 1 h. (**C**) Antibody conjugated nanoliposome formulation at concentration 50 µg/mL for 2 h. (**D**) Antibody conjugated nanoliposome formulation at concentration 100 µg/mL for 2 h.

An in vitro cellular uptake study was also conducted for tamoxifen citrate loaded nanolipid vesicles conjugated with antibody (FITC anti-human CD 340 (erbB2/HER-2)) in MDA-MB-453 (ER-negative) breast cancer cells for different concentration, such as 50 and 100 µg/mL for 1 and 2 h. Figure 9 revealed that tamoxifen citrate nanolipid vesicles penetrated the cell membrane. At higher concentrations, more lipid vesicles were observed in the cells assessed by confocal microscopy images.

It was found that anticancer drug-loaded nanocarriers penetrated and distributed in the MCF-7 cells as well as MDA-MB-453 cells. These results confirm that estrogen receptor (ER)-positive breast cancer cells, as well as ER-negative breast cells, were able to internalize anticancer drug-loaded nanolipid vesicles.

### 3.3. Apoptosis Assay by FACS Analysis

**Figure 10:**
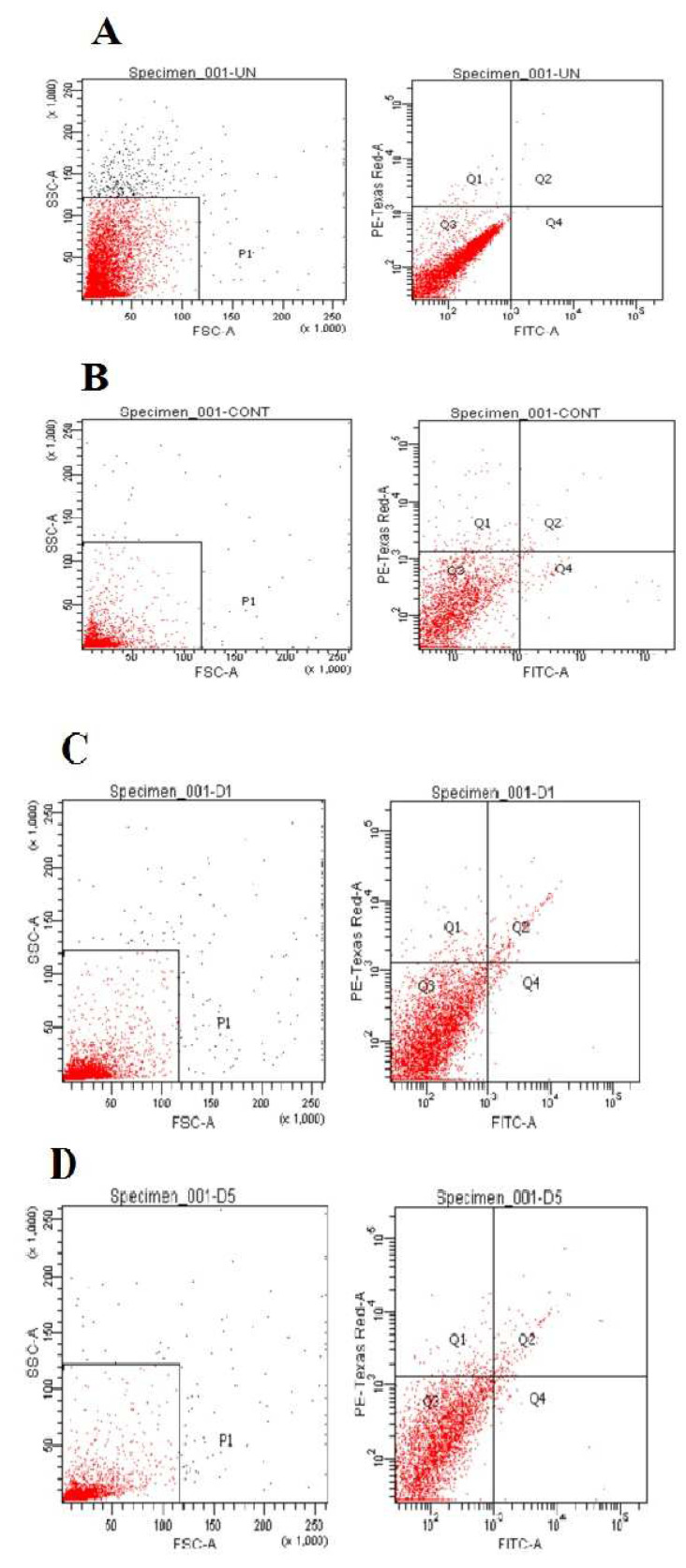
Representative FACS analysis of treated cells (**A**) control cells, (**B**) free drug solution, (**C**,**D**) tamoxifen citrate loaded nanolipid vesicles of 50 and 100 µM, respectively, staining with Annexin V/PI.

A fall in the forward scatter (FSC) signal and an increase in the side scatter (SSC) signal indicate cell death. In Figure 10A, the FSC vs. SSC dot plot from the point of cell analysis showed the basic morphological information of the cells. A flow cytometry study showed that 95.9% of cells were viable, 0.1% were apoptotic, 0.4% were necrotic and there were no preapoptotic cells present. Figure 10B revealed that 90.9% were viable cells, 0.6% were apoptotic cells, 1.3% were preapoptotic and 2% of necrotic cells were present for the free drug solution.

Figure 10C indicates that the percentage of preapoptotic cells and apoptotic cells treated with the liposomal formulation were 1.0% and 1.8%, respectively, while viable cells and necrotic cells were 93.1% and 1.3%. Figure 10D showed that upon liposomal treatment (TNL-PE), the percentage of preapoptotic cells and apoptotic cells in the liposomal formulation were 1.0% and 2.4%, respectively, and 92.3% of viable cells and 2.0% of necrotic cells were present.

MCF-7 cell lines were visualized under flow cytometric analysis after staining of phosphatidylserine translocation with FITC-annexin V in combination with propidium iodide. Figure 10C shows apoptotic cells and necrotic cells (red stain) when exposed to the free drug solution and drug-loaded liposomes. When treated with the liposomal formulation, the number of apoptotic and preapoptotic cells was found to increase. This suggests that liposomal treatment of the drug would be more beneficial for the treatment to manage the disease as compared to the free drug treatment.

## 4. Discussion

The nanolipid vesicles showed smooth spherical shapes with nanosize dimensions in their lyophilized state and were homogeneously scattered. Conjugation of antibody with phosphoethanolamine as a linker molecule in the phospholipid bilayer surface was found to extend the diameter size. The presence of PE within the acyl chains of vesicles might vary the packing density of the acyl chain, leading to the result of the enhancement of the diameter.

The average diameter of TEM and cryo-TEM images showed a higher value as compared to the lyophilized formed as shown in FESEM photographs. The diameter of the nanolipid vesicles observed within the FESEM photographs was much smaller (<50 nm) than the results (>50 nm) of TEM and cryo-TEM photographs, however, they were within 100 nm size ranges. This can be probably due to hydration and swelling of the particles in aqueous media during TEM and cryo-TEM analysis [30].

The nanoliposomes were examined by TEM images (Figure 2). The inner core of the nanoliposome contained drug, and therefore the core was surrounded by a phospholipid bilayer. The TEM images further showed the surface morphology of the vesicle to be smooth and non-porous. The skinny, darker and cylindrical spots showed the presence of cholesterol on the bilayer membrane. Phosphoethanolamine (PE) incorporation showed the presence of many polygonal particulate PE on the bilayer surface of lipid vesicles, which is tagged with antibody. The hydrodynamic diameter of the nanosized vesicles in an aqueous environment was about one micron [31,32]. The TEM data have provided evidence that the lyophilized nanoliposome containing tamoxifen citrate is often reconstituted in an aqueous medium without any leakage within the bilayer membrane, suggesting the physical stability of the formulation in an aqueous environment.

The cryo-TEM images revealed that mostly the unilamellar vesicles’ spherical shape had intact lamellarity. They consisted of a lipid bilayer surrounding an aqueous core. The lyophilized preparation was suspended in water for a longer time for cryo-TEM analysis than the particle size measurement. The hydrodynamic diameters were enhanced, and the size became larger. The dark membrane of the spherical core and the few dark spaces on the inner side of the core indicate the presence of anticancer drugs in the vesicular membrane and the few suspended drug particles in the core when the aqueous layer was entrapped inside the vesicles.

EDX analysis (Figure 5) revealed that the different values of the weight percentage and atomic (Table 1) percentage of the reported elements were due to the presence of antibody labeled in modified nanolipid vesicles. The shifting of wavelength (Figure 4) for different fluorescent color intensities (blue to red) in both two absorption maxima (λ_max_ 494 nm and λ_max_ 525 nm) indicates the presence of antibody which was labeled in modified nanolipid vesicles.

Percentages of drug loading and loading efficiency values were almost similar for both the formulations. The experimental value indicated that after conjugation or labeling with antibody, the quantity of drug entrapment in the inner core of lipid vesicles does not vary. The polydispersity index (PDI) is usually representative of indicators of particle size (in terms of diameter) distribution in colloidal systems [33,34]. The significance of this parameter influences the physical stability and the PDI value showed the heterogenicity of particle sizes in the experimental formulation. In the present study, the PDI values of the experimental formulations were ≤0.15, suggesting that the experimental nanolipid vesicles were physically stable, uniformly dispersed and had less possibility of agglomeration while in suspension [35].

The zeta potential value of the nanolipid vesicles is an important parameter which determines the surface charge and also influences the physical stability of the particles in suspended form. The high zeta potential values indicate a high electrical phenomenon on the surface of the drug-loaded nanolipid vesicles, which may create strong repellent forces within vesicles and prevent aggregation of the nanocarriers in solution [36]. The zeta potential value of nanolipid vesicles during this study was negative due to the presence of terminal carboxylic groups within the soyalecithin. For colloidal dispersion, zeta potential values within +30 mV and −30 mV are considered as stable suspensions [36,37].

The cellular uptake and subcellular distribution of the nanocarriers, as seen by using the confocal laser scanning microscopy, were predominantly concentration-dependent. FITC nanoliposomes also penetrated the cell membrane of MCF-7, likewise for the MDA-MB-453 cells. These results confirm that nanoliposome might be well internalized by estrogen receptor (ER)-positive carcinoma cells and ER-negative breast cells to produce the expansion inhibitory effect of tamoxifen citrate [38]. However, carcinoma cells containing estrogen receptors are more tuned into antiestrogen treatment and have a more robust prognosis than estrogen receptor-negative tumors.

Flow cytometric analysis with annexin V and propidium iodide staining was performed to detect apoptosis by targeting the loss of phospholipid asymmetry within the semipermeable membrane. MCF-7 cells were analyzed by flow cytometry after the staining of phosphatidylserine translocation with FITC-annexin V together with propidium iodide. Nanoliposomal formulation treatment in cells showed more apoptotic cells than the free drug-treated cells. Data suggest that the drug-loaded liposomal formulation would be more beneficial for the treatment of cancer chemotherapy compared to the free drug treatment.

**Figure 11:**
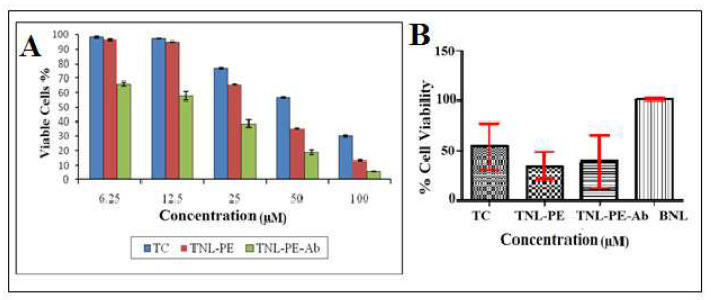
(**A**) Comparison of cell viability percentage between tamoxifen citrate loaded nanolipid vesicles with phosphoethanolamine (TNL-PE), antibody conjugated tamoxifen citrate loaded nanolipid vesicles (TNL-PE-Ab) and free drug solution (TC) on MCF-7 cells for 48 h. (**B**) Percentage of cell viability by statistical data for free drug (TC), TNL-PE, TNL-PE-Ab and blank nanolipid vesicles (BNL).

The toxic effect of the antibody conjugated nanoliposomal formulation (TNL-PE-Ab) decreased cell viability from 66.16 ± 1.30% to 5.71 ± 0.18%. Figure 11 reveals that the cytotoxic effect of TNL-PE-Ab against MCF-7 cells was on top of the TNL1 and TNL-PE formulations.

The liposomes utilized in this study were formed by using phosphatidylethanolamine (PE) as a linker molecule which was conjugated with FITC labeled antibody. The amine-functionalized lipid vesicles containing soyalecithin, cholesterol and PE at different molar ratios in the vesicular formulations are reactive to amine-containing ligands using a linker. A variety of antibodies have been coupled to the surface of the nanocarrier using this crosslinking approach. The glycerol phosphate head group of PE after modification with the amine group influences a perfect function for activation as well as coupling for targeting molecules [39,40]. It has been shown that the accumulation of the targeted liposomes in carcinoma cells (MCF-7 and MDA-MB-453) was over the observed accumulation with nontargeted (without antibody attached) lipid vesicles. Further, the conjugation of specific antibody to the outer surface of liposomes would tend to focus on the relevant specific cells. The developed formulation (lyophilized from) should be kept in a refrigerated condition and the formulation should be reconstituted before use.

In the present study, all experimental data of the nanovesicular formulation indicate that there was no chemical interaction between the pure drug and all other excipients, except in the case of the conjugation reaction that passed off between PE and soyalecithin.

## 5. Conclusions

Tamoxifen citrate loaded nanolipid vesicles were developed by using phosphoethanolamine (PE) as a linker molecule in the phospholipid bilayer in a simple thin-film hydration technique. Average diameters (z-average) of the nanoliposomes were within nanosize dimensions at the lyophilized state, with a negative surface charge (zeta potentials), smooth surface and were homogeneously distributed. The vesicles had an intact structure with no membrane leakage and maintained vesicles lamellarity. The results suggest that tamoxifen citrate loaded nanoliposomes may be beneficial to provide better therapeutic activity because of their smaller size which leads to the “enhanced permeability and retention effect” in malignant cells.

Further, antibody conjugated nanolipid vesicles loaded with anticancer drugs, such as tamoxifen citrate showed predominant cellular uptake in MCF-7 cells as well as in MDA-MB-453 cells. This formulation was developed for active targeting by attaching the specific antibody (FITC anti-human CD 340 (erbB2/HER-2)) to the linker molecules (PE) present on the phospholipid bilayer surface. The antibody conjugated nanoliposome showed a faster and better uptake of them by MCF-7 (estrogen receptor-positive) cancer cells as compared to the treatment of the MDA-MB-453 (estrogen receptor-negative) cancer cells, indicating preferential uptake and suggesting the possibility of their success in the breast cancer cell targeting. FACS analysis showed that the number of apoptotic and preapoptotic cells was found to increase by using the experimental formulation. This suggests that liposomal treatment of the drug would be more beneficial for the treatment to manage the disease as compared to the free drug treatment.

In the future, preferential cellular uptake for different breast cancer cell lines and their quantitative comparison study can be conducted. Further studies are warranted to investigate the efficacy of the antibody conjugated liposome for breast cancer animal models in vivo. However, all experimental data of this present work suggest that the antibody labeled modified nanolipid vesicles can be beneficial for breast cancer chemotherapy.

## Figures and Tables

**Table 1:** Weight % and atomic % of elements in various nanoliposomes.

	Carbon		Oxygen		Phosphorous	
	TNL-PE	TNL-PE-Ab	TNL-PE	TNL-PE-Ab	TNL-PE	TNL-PE-Ab
% Weight	37.58	34.04	51.45	49.03	10.97	16.92
% Atomic	46.71	43.97	48.00	47.55	5.29	8.48

**Table 2:** Zeta potential, poly dispersity index (PDI), mobility and conductivity of nanolipid vesicles.

Formulations	Zeta Potential	PDI *	Mobility	Conductivity
	(mV) *		(μmcm/Vs) *	(MS/cm) *
TNL-PE	−65.2 ± 0.77	0.086 ± 0.06	−5.112 ± 0.06	1.201 ± 0.179
TNL-PE-Ab	−66.8 ± 2.61	0.150 ± 0.07	−5.238 ± 0.20	1.466 ± 0.316

Values represent mean ± SD (*n* = 3). Statistical significance evaluations represented on the table by asterisks (* *p* < 0.05 at 95% confidence interval).

**Table 3:** Percentage of theoretical drug loading, percentage of practical drug loading and loading efficiency of nanolipid vesicles.

Formulation	Theoretical Drug	% Loading	Loading Efficiency
Code	Loading (%) *	(Mean ± SD, *n* = 3)	(% *w*/*w*) (Mean ± SD, *n* = 3)
TNL-PE	5.26	3.13 ± 0.08	90.97 ± 2.34
TNL-PE-Ab	4.76	3.46 ± 0.58	90.13 ± 15.25

Theoretical drug loading (%) = (Amount of drug/Amount of drug + excipients) × 100. Values represent mean ± SD (*n* = 3). Statistical significance evaluations represented on the table by asterisks (* *p* < 0.05 at 95% confidence interval).
